# High-Altitude Hypoxia Decreases Plasma Erythropoietin Soluble Receptor Concentration in Lowlanders

**DOI:** 10.1089/ham.2019.0118

**Published:** 2020-03-17

**Authors:** Gustavo Vizcardo-Galindo, Fabiola León-Velarde, Francisco C. Villafuerte

**Affiliations:** ^1^ Laboratorio de Fisiología Comparada, Departamento de Ciencias Biológicas y Fisiológicas, Facultad de Ciencias y Filosofía, Universidad Peruana Cayetano Heredia, Lima, Perú.; ^2^ Unidad de Transporte de Oxigeno, Instituto de Investigaciones de la Altura (IIA), Universidad Peruana Cayetano Heredia, Lima, Perú.

**Keywords:** erythropoietin, high altitude, soluble erythropoietin receptor

## Abstract

***Background:*** The soluble form of the erythropoietin (Epo) receptor (sEpoR) is an endogenous antagonist of Epo. Decreasing plasma sEpoR increases free Epo, thereby increasing the availability of the hormone. In humans, short-term intermittent normobaric hypoxia exposure reduces sEpoR concentration in plasma. However, whether similar changes occur during continuous hypoxia, such as during high-altitude exposure with ongoing acclimatization, is yet unknown. Therefore, this study aimed to characterize the time-course concentration profile of sEpoR, and also of Epo, reticulocyte count (RC), and hematocrit in healthy lowlanders during 4 days at high altitude.

***Methods:*** Twenty-two men residents at sea level traveled by road (∼7 hours) from Lima to Cerro de Pasco (4340 m) for 72 hours. Oxygen saturation as measured by pulse oximetry (SpO_2_), heart rate, systolic and diastolic blood pressure, Lake Louise Score, sEpoR, Epo, RC, and hematocrit were evaluated every 12 hours, starting 12 hours before the ascent.

***Results:*** Plasma sEpoR decreased by 19% and remained below baseline values throughout high-altitude exposure. In parallel, Epo levels increased during the first hours, reaching a peak at 48 hours, and then progressively decreased until 72 hours. As a result, the Epo-to-sEpoR ratio (Epo/sEpoR) remained significantly elevated compared with baseline values. RC increased linearly until the end of the protocol, and hematocrit only showed a marginal increase.

***Conclusion:*** Our results show that high-altitude hypoxia causes a significant and stable reduction of plasma sEpoR concentration within the first 24 hours, whereas plasma Epo constantly decreases after having reached a maximum by 48 hours. This simultaneous change leads to a relatively high Epo/sEpoR after 72 hours at high altitude. The early increase in hematocrit likely relates to hemoconcentration, but the steady increase in RC reflects a sustained erythropoietic drive that will lead to elevate hematocrit to a new steady state after acclimatization.

## Introduction

Increased erythropoiesis is probably the best known physiological response to chronic hypoxia and a hallmark of high-altitude acclimatization. Hypoxia induces the expression of the glycoprotein hormone erythropoietin (Epo), which mediates the increase in red blood cell (RBC) production. Once in the bloodstream, Epo acts on the bone marrow and promotes the survival, proliferation, and differentiation of erythroid progenitors by binding to its receptor (EpoR) (Koury and Bondurant, 1988, 1990; Broudy et al., 1991; Kelley et al., 1994; Silva et al., 1996). Although Epo is expressed in several tissues, the cortical peritubular fibroblasts in the kidney are the primary site of Epo production, and responsible for its increased concentration in blood during hypoxia (Lacombe et al., 1988; Eckardt et al., 1989; Tan et al., 1992; Bachmann et al., 1993; Marti et al., 1996).

After acute hypoxic exposure, plasma Epo concentration increases within 1.5–2 hours, reaches maximal values within the first 48 hours, and decreases after that despite the sustained hypoxic stimulus. This early decrease occurs before red cell mass, and thus arterial oxygen content increases significantly (Faura et al., 1969; Abbrecht et al., 1972; Milledge and Cotes, 1985). However, the erythropoietic process continues and reaches a steady-state at a substantially higher hematocrit level despite circulating Epo concentration close to sea-level values. This phenomenon is known as the “Epo paradox” (Bärtsch and Milledge, 2014) and implies that only a marginally increased plasma Epo concentration is required to support continued erythropoiesis and to sustain erythropoietic drive under chronic hypoxic conditions. Because the half-life of RBCs is ∼3 months, their rate of destruction results much slower than their rate of production under this long-term altitude exposure, and therefore a higher steady-state hematocrit value is maintained. The increased RBC production rate is achieved by an enhanced erythropoietic sensitivity, which includes the upregulation of membrane EpoR and antiapoptotic factors in erythroid progenitors in the bone marrow (Zon et al., 1991; Ferreira et al., 2005; Zhang et al., 2012). A soluble form of the Epo receptor (sEpoR) has been suggested to modulate the action of plasma Epo before it reaches its target receptor in tissues (Sawada et al., 1987; Nagao et al., 1992; Baynes et al., 1993; Harris and Winkelmann, 1996). Soluble EpoR competes with EpoR to bind Epo, thereby limiting the ability of Epo to bind EpoR. Thus, decreasing plasma sEpoR concentration increases free Epo concentration and therefore increases its availability.

Brugniaux et al. (2011) showed that men exposed to intermittent normobaric hypoxia for 8 days (2-minute hypoxia at P_ET_O_2_ ∼45 mmHg, followed by 2-minute reoxygenation, 6 hours/day) showed significant downregulation of plasma sEpoR from the first day, reaching a 70% reduction by the second day. After reaching its minimum concentration, decline increases by 24%, to then remain stable until the end of the protocol. In parallel, Epo concentration increased by 50% on the second day, and remained elevated 36% from baseline by day 4, to then return to baseline values by day 8 (Brugniaux et al., 2011). However, it is possible that the decline and later partial recovery of sEpoR levels are consequence of the intermittency and short duration of hypoxic exposure, which does not allow for an acclimatization process. Whether these changes occur with a similar or different pattern during short-term high-altitude exposure with ongoing acclimatization, is yet unknown.

We tested the hypothesis that plasma sEpoR concentration decreases during high-altitude hypoxia and remains consistently low during the early acclimatization phase. Therefore, this study aims to characterize the time-course concentration profile of sEpoR in healthy lowlanders at sea level and during 4 days at high altitude.

## Methods

The study was approved by the Institutional Ethics Committee of Universidad Peruana Cayetano Heredia (Lima, Peru). All participants received a detailed explanation of the study procedures and signed an informed consent form.

### Study participants

Twenty-two men, residents at sea level, 19–52 years old, were recruited for the study to travel by road (∼7 hours) from Lima (150 m) to Cerro de Pasco (4340 m, barometric pressure = 456 mmHg) for 72 hours. Exclusion criteria included lung disease, anemia, cardiovascular and/or metabolic disorders, heavy smoking, and ongoing medical treatments. Participants underwent a clinical screening examination, answered general health and Lake Louise Score (LLS) questionnaires, and hematocrit was determined from duplicate microcentrifuged blood samples obtained from a fingertip capillary blood draw. In addition, oxygen saturation as measured by pulse oximetry (SpO_2_) and heart rate (HR) were measured with a pulse oximeter (Oximax N-560; Nellcor, Minneapolis, MN), and blood pressure (BP) with an oscillometric validated device (A&D UA-767Plus; A&D). Twelve hours before departure (−12 hours), the first set of measurements was taken. A small blood sample was taken for hematocrit determination, and two 3 mL blood samples were taken from the antecubital vein in ethylenediaminetetraacetic acid (EDTA)-coated tubes. One tube was used for reticulocyte count (RC), and the second was centrifuged at 3500 rpm for 10 minutes. Plasma was used for Epo, sEpoR, iron, transferrin, and ferritin determination. The estimation of plasma volume was calculated from hematocrit values obtained from venous blood samples. The following morning, after 12 hours, the final set of measurements at sea level was taken (0 hour). After traveling by road to Cerro de Pasco, and once 12 hours have passed since morning measurements at sea level, the first set was taken at altitude (12 hours). The same procedure was repeated every 12 hours until the last sample at 72 hours in Cerro de Pasco was taken. Plasma samples were stored at −20°C, transported to Lima in a liquid N_2_ dry shipper, and finally stored at −80°C until analysis.

### Reticulocyte count

RC was performed by standard optical microscopy using brilliant cresyl blue. In brief, 150 μL of blood and 150 μL of the dye were gently mixed in an EDTA-coated tube and incubated for 5 minutes at 37°C. Immediately after, 50 μL of the mix were extended on a microscope slide and let to dry at room temperature (Koepke and Koepke, 1986). Cell count was performed at 100 × . The number of reticulocytes per 1000 cells was divided by the number of erythrocytes observed and multiplied by 100. Results are expressed as percentage.

### Plasma Epo and sEpoR determination

Total plasma Epo and sEpoR were quantified using specific sandwich enzyme-linked immunosorbent assay kits (DRG International, Inc., Springfield, NJ, and USCN Life Science, Inc., Houston, TX; respectively). The standard sample storage and analysis procedure described by the manufacturer was followed for each kit. The minimum detectable concentration of Epo and sEpoR for these assays were typically <1.1 mUI/mL and 0.57 ng/mL, respectively. Antibodies for the receptor provided in the kit were previously tested for specific recognition by immunohistochemical staining and western blot, and measured total sEpoR concentration by binding to both free and bound sEpoR. Detection of sEpoR in the assay was verified using lyophilized soluble receptor isolated from human plasma at a concentration of 0.79 ng/mL as a positive control provided by the manufacturer. The Epo-to-sEpoR ratio (Epo/sEpoR) was calculated for each time point.

### Statistical analysis

Normality of distribution and homogeneity of variance of variables was assessed before any further comparison to choose an adequate statistical analysis test. Multiple comparisons were performed using repeated-measures one-way analysis of variance test when data met parametric requirements, or Friedman test if otherwise. Tukey's or Dunn's tests were used for multiple *post hoc* comparisons, respectively. GraphPad software version 6 was used for statistical analysis (GraphPad Software, La Jolla, CA).

## Results

**FIG. 1.**
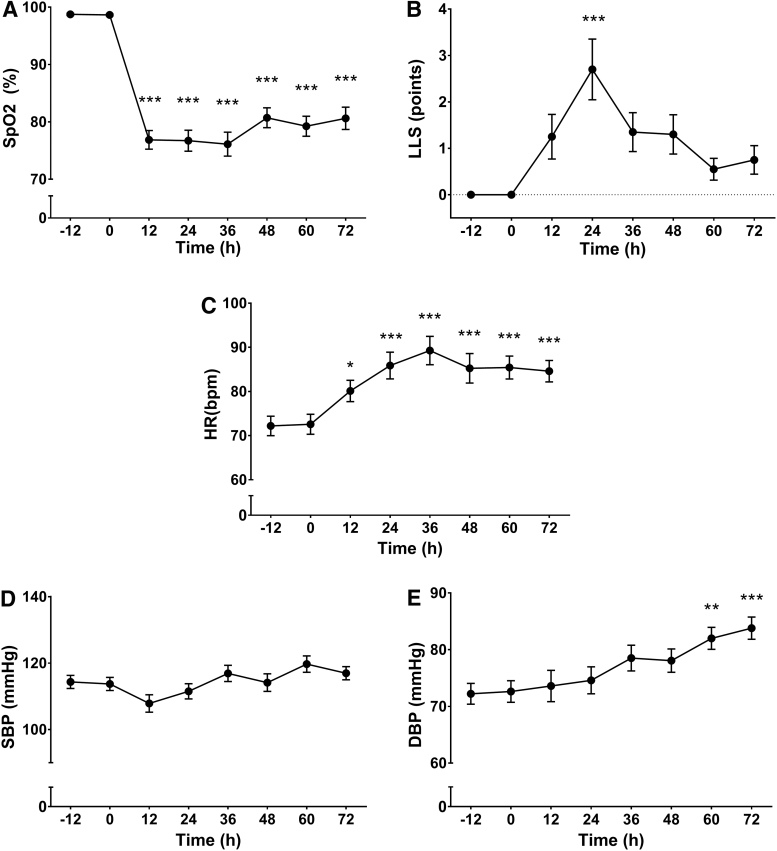
Time-course of general physiological parameters in healthy lowlanders (*n* = 20) during high-altitude (4340 m) hypoxic exposure. SpO_2_ **(A)**, LLS **(B)**, HR **(C)**, SBP **(D)**, and DBP **(E)**. Each time point was compared with its baseline value at sea level (0 hour). Values are expressed as mean ± SE. *p < 0.05, **p < 0.01, and ***p < 0.001. DBP, diastolic blood pressure; HR, heart rate; LLS, Lake Louise score; SBP, systolic blood pressure; SE, standard error; SpO_2_, oxygen saturation as measured by pulse oximetry.

Characteristics of participants and baseline physiological measurements are summarized in Table 1. Figure 1 shows the time-course of changes in SpO_2_, LLS, HR, systolic blood pressure (SBP), and diastolic blood pressure (DBP) during the stay at high altitude. Changes in physiological variables observed on the first measurement at high altitude (12 hours) are the result of an ascending ∼7 hours road trip and not of continued exposure to 4340 m.

**Table 1.** Characteristics of Study Participants

Age, years	32.7 ± 1.9
Weight, kg	71.3 ± 2.8
Height, m	1.7 ± 0.1
BMI, kg/m^2^	26 ± 0.8
Hct, %	43.6 ± 0.5
SpO_2_, %	98.7 ± 0.2
HR, bpm	72.5 ± 2.3
SBP, mmHg	113.7 ± 1.9
DBP, mmHg	72.6 ± 2.2

Values are expressed as mean ± SE.

BMI, body mass index; DBP, diastolic blood pressure; Hct, hematocrit; HR, heart rate; SBP, systolic blood pressure; SpO_2_, oxygen saturation as measured by pulse oximetry.

**FIG. 2.**
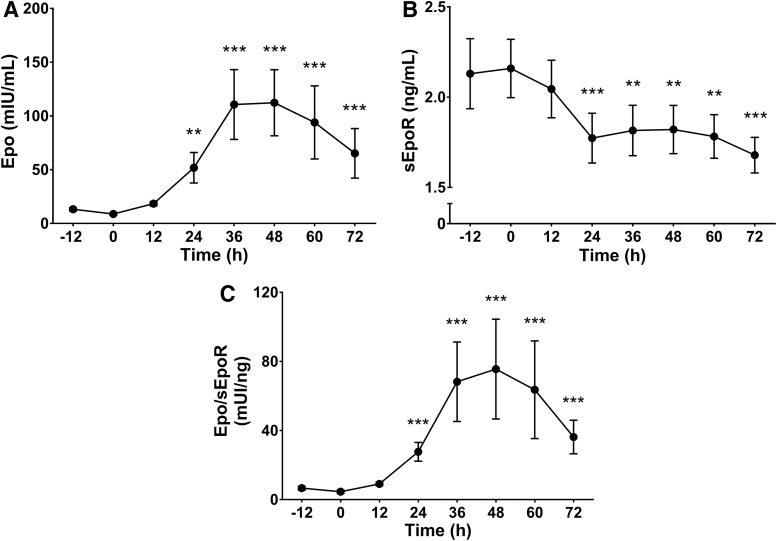
Plasma Epo, sEpoR, and Epo/sEpoR during high-altitude exposure. Plasma Epo **(A)** and sEpoR **(B)** concentration were measured every 12 hours over a 72 hours period at high altitude, starting the night before the ascent to Cerro de Pasco (4340 m). The Epo-to-sEpoR ratio was calculated for each time point **(C)**. Each measurement was compared with its baseline value at sea level (0 hour). Values are expressed as mean ± SE. **p < 0.01, and ***p < 0.001. Epo, erythropoietin; sEpoR, soluble form of the erythropoietin receptor.

At sea level, plasma sEpoR and Epo showed no differences between the night before ascending (−12 hours) and the morning of the ascent (0 hour). Twelve hours after morning measurements and once at Cerro de Pasco (12 hours), Epo concentration increased significantly by 24 hours (590%, *p* < 0.01) and peaked at 48 hours (1320%, *p* < 0.001), to thereafter decrease progressively, remaining above baseline after 72 hours (Fig. 2A). In parallel, plasma sEpoR concentration started to fall from 12 hours, with a reduction of 17% by 24 hours (*p* < 0.001), and a maximum decline of 19% at 72 hours (*p* < 0.001) (Fig. 2B). The Epo/sEpoR reached a peak value at 48 hours (1584%, *p* < 0.001), and after that decreased progressively reaching a minimum at 72 hours, but remained 640% above baseline (*p* < 0.001) (Fig. 2C).

**FIG. 3.**
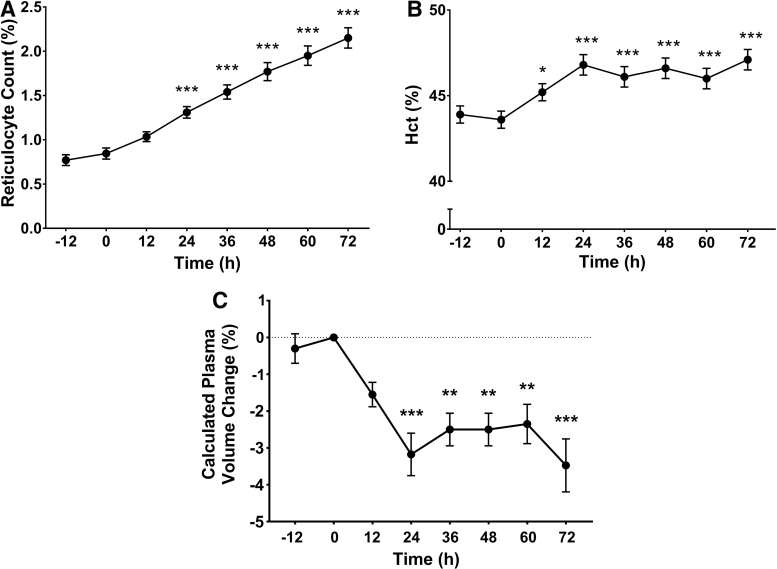
RC, hematocrit, and calculated plasma volume change during high-altitude exposure. RC **(A)** and Hct **(B)** were measured every 12 hours over a 72 hours period at high altitude, starting the night before the ascent to Cerro de Pasco (4340 m). Plasma volume change was calculated and expressed as percentage variation for each time point **(C)**. Each measurement was compared with its baseline value at sea level (0 hour). Values are expressed as mean ± SE. *p < 0.05, **p < 0.01 and ***p < 0.001. Hct, hematocrit; RC, reticulocyte count.

RC increased significantly from arrival and continued to increase linearly until the end of the protocol (Fig. 3A). Hematocrit increased slightly from arrival and continued stable for the remaining 60 hours at 4340 m (Fig. 3B). Calculated plasma volume fell significantly by 12 hours, and continued to decrease until the end of the protocol (Fig. 3C). Among iron status markers, only ferritin showed an increase at 24 hours from baseline, whereas iron and transferrin showed no differences during high-altitude exposure (Table 2). Serum iron, ferritin, and transferrin remained within the normal range (WHO, 2007, 2011).

**Table 2.** Iron Profile

	0 Hour	24 Hours	48 Hours
Serum iron, μg/dL	90.2 ± 5.68	86.4 ± 4.35	96.2 ± 6.58
Serum ferritin, ng/mL	117.9 ± 12.26	134.5 ± 12.77^***^	122.4 ± 12.04
Serum transferrin, mg/mL	254.1 ± 7.54	268.4 ± 8.43^*^	270 ± 8.15^**^
Transferrin saturation, %	25.3 ± 1.61	22.9 ± 0.94	25.4 ± 1.67

Iron status profile compared to baseline values at sea level (0 hours). Values are expressed as mean ± SE, ^*^p < 0.05, ^**^p < 0.01 and ^***^p < 0.001.

## Discussion

This is the first study to report changes in the circulating pair sEpoR-Epo and erythropoietic markers during the early phase of the acclimatization process to high-altitude hypobaric hypoxia. We show that in parallel to the well-known hypoxia-induced increase in circulating Epo concentration, plasma sEpoR levels decrease at high altitude and remain below baseline throughout altitude exposure.

The effect of high altitude on SpO_2_, LLS, SBP, and DBP agrees with findings of previous studies in lowlanders exposed acutely to similar altitudes (Reeves et al., 1993; Beall, 2006; Goetze et al., 2013). Despite the age range of participants, SpO_2_, LLS, SBP, and DBP values showed strong consistency. Only two participants showed significantly lower SpO_2_ values, and higher LLS at 24 hours compared with the rest of the group. The initial decrease in SpO_2_, recovered partially after 36 hours, reflecting an ongoing acclimatization process. In a similar way, HR, which showed an increase during the first hours of high-altitude exposure, reached a plateau after 36 hours, also showing the progress of altitude acclimatization. This is also observed in the decrease of the typical high-altitude symptomatology quantified through LLS, which usually peaks after 2 days at altitude, and then decreases consistently to almost baseline after 60 hours. Although variations in iron status markers are uncommon during short altitude exposure (Piperno et al., 2011; Goetze et al., 2013; Bärtsch and Milledge, 2014), the finding of increased serum ferritin at 24 hours, which coincides with peak LLS, might indicate an elevated inflammatory state in some of the participants (Hartmann et al., 2000; Nicolas et al., 2002; Merle et al., 2007; Imray et al., 2010). Our study confirms the downregulating effect of hypoxia on sEpoR shown by previous studies in humans and animals. Soliz et al. (2007) showed that 3-day normobaric hypoxia (10% O_2_) downregulated the expression of sEpoR by 62% in brain tissue of mice. In addition, the only study on sEpoR in humans under short-term intermittent hypoxia showed that the receptor concentration in plasma shows an initial decrease and then slight recovery by the end of hypoxic exposure. The study used short hypoxic bouts (2 minutes at P_ET_O_2_ ∼45 mmHg) followed by 2 minutes of reoxygenation for 6 hours/day.

In comparison, our results show that at high-altitude and under similar hypoxic conditions (P_ET_O_2_ ∼45 mmHg), plasma sEpoR shows a lesser decrease, but more stable values without signs of recovery. The comparatively more pronounced effect of intermittent hypoxia exposure on plasma sEpoR levels is probably the result of the short severe hypoxic bouts leaving no place for acclimatization. Of course, depending on the intermittent hypoxia paradigm, and the time allowed for acclimatization, less or more pronounced effects on plasma sEpoR could be expected. A steady lower plasma sEpoR might be of importance during acclimatization, not only for sustaining erythropoietic drive because of its potential modulatory effect on Epo availability in chronic hypoxia, but also because of its impact on ventilatory acclimatization (Soliz et al., 2005, 2007; Gassmann et al., 2009; Brugniaux et al., 2011). Epo availability can be assessed by changes in the Epo/sEpoR (Villafuerte et al., 2014, 2016). In this study, plasma Epo/sEpoR values and Epo concentration followed a similar variation pattern, but the percentage of change from baseline of the former was always greater because of the decrease in sEpoR, which could result in a slight increase in Epo availability. Although 72 hours of high-altitude exposure were not enough for plasma Epo to return close to baseline levels, Epo/sEpoR remained comparatively higher (640% vs. 485%, respectively). It would be expected that Epo/sEpoR remains elevated during a longer high-altitude exposure time because of a further decrease in Epo concentration. This in turn would result in a sustained stimulus for erythropoiesis during long-term or chronic hypoxia. The maintenance of erythropoietic drive in our study is reflected in the steady increase of RC from early altitude exposure throughout the end of the protocol, suggesting a constant erythropoietic stimulus despite declining Epo levels.

During this short high-altitude exposure, however, this steady erythropoietic drive is not yet adequately reflected in an increased RBC count because reticulocytes may take from 5 to 7 days before they differentiate into mature erythrocytes and are released into the bloodstream (Connie and Eaves, 1977, 1978; Elliott et al., 2008). For this reason, the slight increase in hematocrit observed in our study is most probably the result of changes in plasma volume. As it has been demonstrated by previous studies, the marginal increase in hematocrit during early high-altitude exposure results from hemoconcentration because of a contraction of plasma volume most possibly as consequence of dehydration from hyperventilation and increased diuresis (Koller et al., 1991; Modesti et al., 2006; Siebenmann et al., 2017). Our results show a calculated reduction of ∼4% in plasma volume, which is within the range recently reported by Siebenmann et al. (2017) of a decrease ∼7% after 5 days at 4000–5000 m.

In conclusion, our results show that high-altitude hypoxia causes a significant and stable reduction of plasma sEpoR concentration within the first 24 hours, whereas plasma Epo decreases continuously after having reached a maximum by 48 hours. This simultaneous change leads to a relatively high Epo/sEpoR after 72 hours at high altitude. The early increase in hematocrit likely relates to hemoconcentration, but the steady increase in RC reflects a sustained erythropoietic drive that will lead to elevate hematocrit to a new steady state after acclimatization.
